# Association of residential greenspace with incident depression: a prospective cohort study

**DOI:** 10.3389/fpubh.2026.1897674

**Published:** 2026-09-30

**Authors:** Chengfang Yang, Tong Lan, Yinghong Zhai, Bin Liu, Fangyuan Hu, Jianbo Ba

**Affiliations:** 1 Naval Medical Center, Naval Medical University, Shanghai, China; 2 PLA Rocket Force Characteristic Medical Center, Beijing, China; 3 Clinical Research Unit, Shanghai Ninth People's Hospital, Shanghai Jiao Tong University School of Medicine, Shanghai, China; 4 Faculty of Military Health Service, Naval Medical University, Shanghai, China

**Keywords:** cohort study, depression, greenspace, restricted cubic spline regression (RCS), UK Biobank

## Abstract

**Objective:**

Depression is a major public health challenge worldwide. We aimed to examine the association between residential greenspace and incident depression in UK Biobank participants.

**Methods:**

We conducted a prospective cohort study of 265,311 UK Biobank participants. Cox regression models were used to examine the association between residential greenspace (300 m/1,000 m buffers) and all-cause depression, adjusting for varying degrees of confounders across 6 sequential models. Restricted cubic spline regression (RCS) was additionally adopted to assess the dose-response relationship between continuous greenspace and depression risk. A series of sensitivity, subgroup and mediation analyses were also conducted.

**Results:**

265,311 participants entered the primary analysis. During the follow-up period, incident all-cause depression occurred in 10,766 participants (4.06%). The mean greenspace percentage was 36.2% (range: 0%−100%) in the 300 m buffer and 46.0% (range: 0%−100%) in the 1,000 m buffer. In a fully adjusted multivariable model (Model 6), the adjusted hazard ratio for per 10% increment in greenspace percentage was 0.988 (95% *CI*, 0.979–0.996) for the 300 m buffer and 0.983 (95% *CI*, 0.974–0.992) for the 1,000 m buffer, corresponding to a 1.2% and 1.7% reduction in depression risk, respectively. Trend test indicated a statistically significant decreasing trend with increasing greenspace levels (*P* < 0.0001). Similarly, RCS displayed an inverse association of greenspace with all-cause depression, wherein higher greenspace percentage was linked to a reduced risk of depression. Results of subgroup and mediation analyses demonstrated consistent findings.

**Conclusion:**

Increased residential greenspace is associated with a lower risk of all-cause depression. Urban planning strategies that enhance green environments may represent a potential approach for reducing depression risk at the population level.

## 1 Introduction

Depression encompasses a diverse array of mental health disorders with manifestations varying in severity and functional impairment based on underlying causes (1). Depression is a multifactorial mental disorder associated with various etiologies, including genetic susceptibility, psychological stress, social environment, and physical health status (2). Depressive disorders mark a leading cause of global disability, characterized by persistent low mood, cognitive impairment, and impaired daily social functioning (3). Depression is an important global public health problem with a high disease burden and limited population-level preventive interventions (4).

Instead of concentrating on individual environmental factors, residential environmental patterns consider overall built and natural surroundings, offering a more comprehensive reflection of potential synergistic effects of environmental components compared to single-factor analyses (5, 6). Several studies have investigated different environmental indicators as preventive interventions for mental disorders (7). There is evidence suggesting that increasing residential greenspace access can improve mental well-being and at least delay the onset of depressive symptoms (8). However, limited evidence is available regarding the psychosocial and environmental pathways that may explain the protective effects of residential greenspace against depression (9, 10).

Indeed, residential environmental conditions can exert profound impacts on mental health, which are reflected in the psychological and social adaptation of residents (11). Accumulating epidemiological evidence has suggested that higher exposure to residential environmental greenspace is associated with better mental health and decreased risk of depressive symptoms (12). As a comprehensive and reliable indicator for quantifying the overall greenness of the living environment, residential greenspace percentage has been consistently associated with a variety of mental health outcomes, such as anxiety, psychological distress, and depression (12). Compared with single-point greenness measurements, greenspace percentage within 300 m and 1,000 m geographic buffers corresponds better to the daily activity range of residents and shows stronger predictive value for mental health. Although previous prospective studies have examined greenspace and depression using different spatial buffers and exposure metrics, evidence from large prospective cohorts that simultaneously considers residential-scale variation and multiple environmental co-exposures remains limited (13, 14). UK Biobank is a large-scale prospective cohort study that recruited approximately 500,000 participants aged 40–69 years from 22 assessment centres across England, Scotland, and Wales between 2006 and 2010 (15). The assessment centres were distributed across different geographic regions, enabling recruitment of participants from diverse residential settings. Therefore, we hypothesized that higher greenspace availability in the residential environment may be associated with a lower risk of incident all-cause depression, which has not been fully clarified in previous studies with comprehensive control of environmental covariates. To fill this research gap, we performed a population-based prospective cohort study using data from the UK Biobank, aiming to examine the associations between environmental greenspace percentage in 300 m and 1,000 m buffers and the subsequent risk of developing all-cause depression.

## 2 Methods

The UK Biobank (https://www.ukbiobank.ac.uk/) represents a large-scale, long-term prospective cohort initiative, featuring rich data on phenotypic traits, environmental exposures and longitudinal health outcomes (15–17). Under an open-access framework, it enables investigators across academic and industrial sectors worldwide to dissect the etiology of common complex physical and mental disorders, by exploring their connections with environmental, lifestyle and sociodemographic factors (18, 19). From 2006 to 2010, around 500,000 volunteers aged 40–69 years were enrolled across 22 assessment centers throughout the United Kingdom (19). All participants completed comprehensive questionnaires, took physical examinations and environmental exposure assessments, and agreed to long-term follow-up as well as linkage with routine health records. The UK Biobank was approved by the relevant research ethics committee (approval number: 11/NW/0382), and all participants provided written informed consent upon enrollment (19). The present study was conducted under UK biobank application number [99709].

### 2.1 Study population

The present study included participants from the UK Biobank cohort. Among the original 502,428 participants, individuals with depression at baseline were excluded (*n* = 41,720). Participants with missing data on key variables were subsequently excluded (*n* = 190,680), as were those with a baseline residential duration of less than 1 year (*n* = 4,717). Finally, 265,311 participants with complete information on greenspace exposure, incident depression, and all covariates were included in the primary analysis (Figure 1).

**Figure 1:**
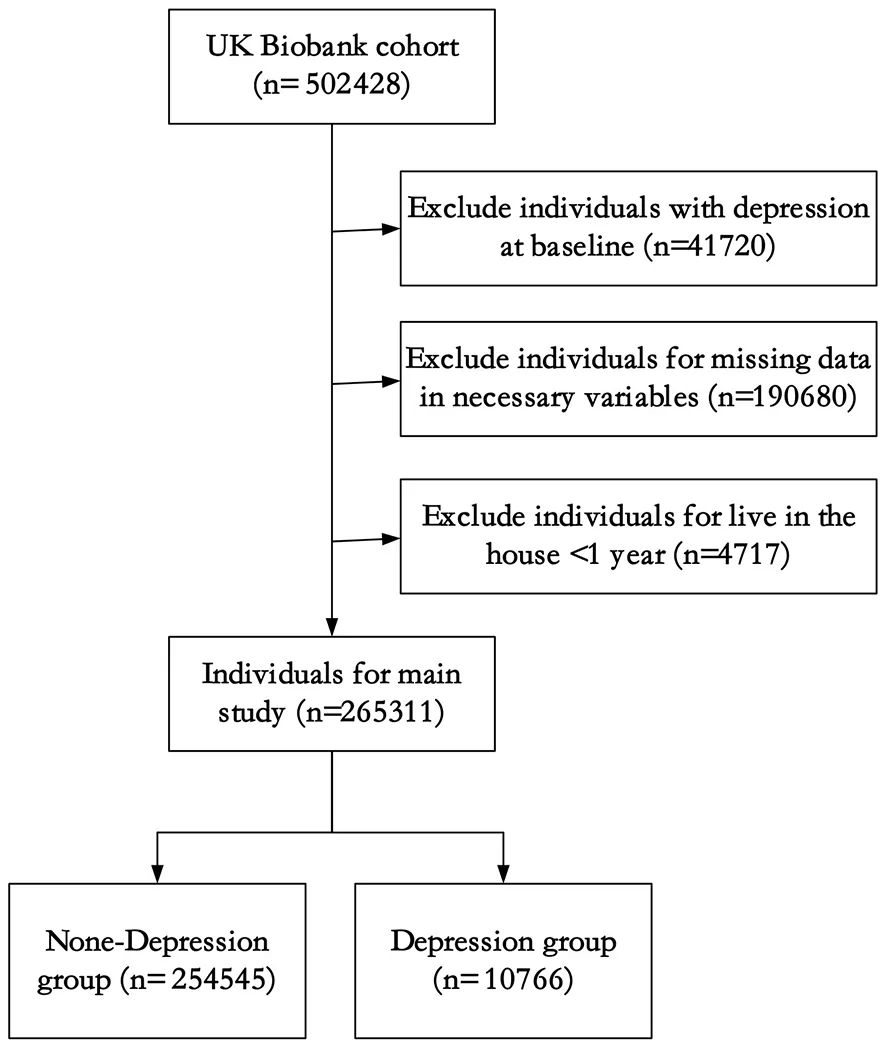
Flow chart of the study.

### 2.2 Assessment of greenspace percentage

Residential greenspace exposure was obtained from the UK Biobank environmental exposure dataset. Greenspace percentage was defined as the proportion of the residential buffer area classified as “Greenspace” among all land-use types. Two circular buffers (300 m and 1,000 m) were generated around each participant's geocoded baseline residential location. The greenspace variables were derived from the Generalized Land Use Database for England (GLUD) 2005, based on land-use information at the 2001 Census Output Area level. For each residential buffer, an area-weighted mean percentage of greenspace was calculated based on the intersecting Census Output Areas. These variables were available for participants whose baseline residential locations were in England. Greenspace percentage within the 300 m and 1,000 m buffers was analyzed as a continuous exposure variable in all analyses (20). Detailed information regarding the derivation of greenspace variables is provided in Supplementary Table S2.

### 2.3 Outcome ascertainment

New-onset all-cause depression was the primary outcome of the present study. The ascertainment of depression in the UK Biobank was defined using the International Classification of Diseases, 10th Revision (ICD-10) codes F32 (depressive episode) and F33 (recurrent depressive disorder) (21). These codes were extracted from the first occurrence dataset (Data Category: 1712) in the UK Biobank. This dataset indicates the initial occurrence of a set of diagnostic codes encompassing a broad spectrum of health outcomes, sourced from self-report, primary care, hospital inpatient data, and mortality records. All eligible individuals were followed up from initial recruitment until the first incident diagnosis of depression, the date of death, or the end (June 1, 2024), whichever came first.

### 2.4 Covariates

Demographic characteristics including age, sex, educational attainment, ethnic origin, and current employment status were collected from the baseline assessment. Lifestyle factors included alcohol drinker status, smoking status, sleep duration, coffee intake, tea intake, and physical activity. Specifically, physical activity was assessed according to a short form of the International Physical Activity Questionnaire (IPAQ) collected during the baseline assessment, and total physical activity was classified into high, intermediate, and low levels in the present study. Body mass index (BMI) was derived from measurements of height (m) and weight (kg) using weight divided by height squared. Health status covariates included history of diabetes, hypertension, cancer, and overall health rating. Environmental co-exposures were obtained from the UK Biobank environmental exposure dataset and linked to participants' baseline residential locations. These variables included traffic noise indicators (24-h, daytime, and nighttime sound levels), particulate matter (PM_2.5 − 10_ μm; 2010), and domestic water quality indicators, including water hardness, calcium concentration, calcium carbonate concentration, and magnesium concentration. Detailed definitions and derivation methods of these environmental variables are provided in Supplementary Table S2.

### 2.5 Statistical analysis

Baseline characteristics of participants were expressed as mean (standard deviation, SD) for continuous variables, and as frequency [percentage, *n* (%)] for categorical variables. Between-group differences in baseline characteristics were compared using Student's *t*-test for continuous variables and chi-square test for categorical variables. The greenspace percentage was analyzed as a continuous variable in all primary analyses.

Cox proportional hazards regression was employed to assess the association between greenspace percentage (300 m and 1,000 m buffers) and incident depression. Greenspace percentage was analyzed as a continuous variable in all primary analyses. Six sequential models were constructed to evaluate the robustness of the associations with increasing adjustment for potential confounders. Model 1 included demographic characteristics; Model 2 additionally incorporated socioeconomic factors; Model 3 further adjusted for lifestyle-related factors; Model 4 included major clinical conditions and overall health status; Model 5 additionally accounted for behavioral and environmental factors; and Model 6 was the fully adjusted model, further including air pollution and water-related environmental factors. Detailed covariate specifications for each model are presented in Table 1. Hazard ratios (HRs) and 95% confidence intervals (CIs) were reported.

**Table 1:** Associations between greenspace and depression incidence in UK Biobank.

Model	Greenspace percentage in 300 m, HR (95% *CI*)	*P*_Cox − regression_	Greenspace percentage in 1,000 m, HR (95% *CI*)	*P*_Cox − regression_
Model 1	0.979 (0.971, 0.987)	<0.0001	0.974 (0.965, 0.983)	<0.0001
Model 2	0.976 (0.968, 0.984)	<0.0001	0.969 (0.960, 0.977)	<0.0001
Model 3	0.984 (0.975, 0.992)	0.0001	0.980 (0.971, 0.988)	<0.0001
Model 4	0.992 (0.983, 1.000)	0.0538	0.989 (0.980, 0.998)	0.0168
Model 5	0.992 (0.983, 1.000)	0.0533	0.989 (0.980, 0.998)	0.0193
Model 6	0.988 (0.979, 0.996)	0.0049	0.983 (0.974, 0.992)	0.0003

Data are presented as hazard ratios (HR) and 95% confidence intervals (CI) per 10% increment in greenspace percentage.

Model 1: sex, age at recruitment, ethnic background and BMI.

Model 2: variables in Model 1 plus employment status and university education.

Model 3: variables in Model 2 plus alcohol drinker status, smoking status and sleep duration.

Model 4: variables in Model 3 plus diabetes, hypertension, cancer and overall health rating.

Model 5: variables in Model 4 plus coffee intake, tea intake, IPAQ activity group, 24 h-sound level, daytime sound level and nighttime sound level.

Model 6: variables in Model 5 plus PM_2.5 − 10_ μm, water hardness (USGS classification), Ca concentration, CaCO_3_ concentration and Mg concentration.

IPAQ, international physical activity questionnaire; USGS, United States Geological Survey (water hardness classification criteria); PM, particulate matter.

Restricted cubic spline (RCS) regression was adopted to determine the dose-response association between continuous greenspace exposure and depression risk in the fully adjusted model. Subgroup analyses were carried out using the fully adjusted model (Model 6) to assess potential heterogeneity in associations between greenspace percentage and depression across strata of key confounders. These included sex, age, BMI, ethnicity, education, employment status, sleep duration, overall health rating, alcohol frequency, physical activity, smoking, diabetes, and hypertension. Mediation analyses were performed to explore the mediating effects of sleep duration and nighttime sound level on the association between greenspace and depression.

We used R (version 4.2.1) for all data analyses and graph plotting. All statistical tests were two-tailed, and *P*-values less than 0.05 were considered statistically significant.

## 3 Results

### 3.1 Baseline characteristics

Among the 265,311 participants included in the primary analysis, 254,545 participants did not develop depression during follow-up, whereas 10,766 participants developed incident depression. Baseline characteristics stratified by incident depression status are presented in Table 2. Most baseline characteristics differed significantly between groups (all *P* < 0.05) except for coarse particulate matter (PM_2.5 − 10_ μm) exposure.

**Table 2:** Characteristics of study population in UK Biobank.

Characteristics	Baseline cohort (*n* = 265,311)	No depression developed (*n* = 254,545) (%)	Developed depression (*n* = 10,766) (%)	*P*-value
**Sex**				<0.0001
Male	129,488 (48.8%)	125,185 (49.2%)	4,303 (40.0%)	
Female	135,823 (51.2%)	129,360 (50.8%)	6,463 (60.0%)	
**Age at recruitment**				0.0043
<60	151,026 (56.9%)	144,754 (56.9%)	6,272 (58.3%)	
≥60	114,285 (43.1%)	109,791 (43.1%)	4,494 (41.7%)	
**BMI (kg/m** ^**2**^ **)**				<0.0001
<18.5	2,438 (0.9%)	2,278 (0.9%)	160 (1.5%)	
18.5–20	4,866 (1.8%)	4,673 (1.8%)	193 (1.8%)	
20–25	84,746 (31.9%)	81,952 (32.2%)	2,794 (26.0%)	
25–30	114,051 (43.0%)	109,735 (43.1%)	4,316 (40.1%)	
≥30	59,210 (22.3%)	55,907 (22.0%)	3,303 (30.7%)	
**Ethnic background**				<0.0001
White	252,509 (95.2%)	242,194 (95.1%)	10,315 (95.8%)	
African	1,767 (0.7%)	1,718 (0.7%)	49 (0.5%)	
Asian	5,317 (2.0%)	5,159 (2.0%)	158 (1.5%)	
Mixed	1,517 (0.6%)	1,445 (0.6%)	72 (0.7%)	
Other ethnic group	4,201 (1.6%)	4,029 (1.6%)	172 (1.6%)	
**University education**				<0.0001
No	171,519 (64.6%)	163,731 (64.3%)	7,788 (72.3%)	
Yes	93,792 (35.4%)	90,814 (35.7%)	2,978 (27.7%)	
**Current employment status**				<0.0001
Employment	160,085 (60.3%)	154,345 (60.6%)	5,740 (53.3%)	
Unemployment	17,216 (6.5%)	15,799 (6.2%)	1,417 (13.2%)	
Retired	88,010 (33.2%)	84,401 (33.2%)	3,609 (33.5%)	
**Alcohol drinker status**				<0.0001
Current	247,705 (93.4%)	238,074 (93.5%)	9,631 (89.5%)	
Previous	8,010 (3.0%)	7,342 (2.9%)	668 (6.2%)	
Never	9,596 (3.6%)	9,129 (3.6%)	467 (4.3%)	
**Smoking status**				<0.0001
Current	24,628 (9.3%)	22,926 (9.0%)	1,702 (15.8%)	
Previous	94,036 (35.4%)	90,024 (35.4%)	4,012 (37.3%)	
Never	146,647 (55.3%)	141,595 (55.6%)	5,052 (46.9%)	
**Sleep duration (h)**				<0.0001
<7	62,184 (23.4%)	58,908 (23.1%)	3,276 (30.4%)	
≥7	203,127 (76.6%)	195,637 (76.9%)	7,490 (69.6%)	
**Diabetes**				<0.0001
No	242,155 (91.3%)	233,101 (91.6%)	9,054 (84.1%)	
Yes	23,156 (8.7%)	21,444 (8.4%)	1,712 (15.9%)	
**Hypertension**				<0.0001
No	165,587 (62.4%)	160,430 (63.0%)	5,157 (47.9%)	
Yes	99,724 (37.6%)	94,115 (37.0%)	5,609 (52.1%)	
**Cancer**				<0.0001
No	202,560 (76.3%)	194,942 (76.6%)	7,618 (70.8%)	
Yes	62,751 (23.7%)	59,603 (23.4%)	3,148 (29.2%)	
**Overall health rating**				<0.0001
Excellent	48,387 (18.2%)	47,434 (18.6%)	953 (8.9%)	
Good	158,653 (59.8%)	153,267 (60.2%)	5,386 (50.0%)	
Fair	49,687 (18.7%)	46,398 (18.2%)	3,289 (30.5%)	
Poor	8,584 (3.2%)	7,446 (2.9%)	1,138 (10.6%)	
**Coffee intake (cups)**				<0.0001
<1	74,638 (28.1%)	71,278 (28.0%)	3,360 (31.2%)	
1	54,790 (20.7%)	52,639 (20.7%)	2,151 (20.0%)	
2	51,323 (19.3%)	49,440 (19.4%)	1,883 (17.5%)	
3	33,152 (12.5%)	31,992 (12.6%)	1,160 (10.8%)	
4	22,532 (8.5%)	21,609 (8.5%)	923 (8.6%)	
5	13,251 (5.0%)	12,711 (5.0%)	540 (5.0%)	
≥6	15,625 (5.9%)	14,876 (5.8%)	749 (7.0%)	
**Tea intake (cups)**				<0.0001
<1	46,056 (17.4%)	43,996 (17.3%)	2,060 (19.1%)	
1	22,997 (8.7%)	22,148 (8.7%)	849 (7.9%)	
2	38,661 (14.6%)	37,221 (14.6%)	1,440 (13.4%)	
3	40,373 (15.2%)	38,870 (15.3%)	1,503 (14.0%)	
4	38,145 (14.4%)	36,679 (14.4%)	1,466 (13.6%)	
5	29,962 (11.3%)	28,728 (11.3%)	1,234 (11.5%)	
≥6	49,117 (18.5%)	46,903 (18.4%)	2,214 (20.6%)	
**IPAQ activity group**				<0.0001
Low	47,182 (17.8%)	44,786 (17.6%)	2,396 (22.3%)	
Moderate	107,456 (40.5%)	103,302 (40.6%)	4,154 (38.6%)	
High	110,673 (41.7%)	106,457 (41.8%)	4,216 (39.2%)	
24 h-sound level, mean (SD)	56.1 (4.28)	56.1 (4.28)	56.2 (4.30)	0.0009
Daytime sound level, mean (SD)	55.4 (4.28)	55.4 (4.28)	55.5 (4.30)	0.0010
Nighttime sound level, mean (SD)	46.6 (4.28)	46.6 (4.28)	46.7 (4.30)	0.0010
PM_2.5 − 10_ μm, mean (SD)	6.42 (0.880)	6.42 (0.879)	6.42 (0.891)	0.9626
Water hardness (USGS classification), mean (SD)	2.48 (1.30)	2.49 (1.30)	2.35 (1.29)	<0.0001
Ca concentration, mean (SD)	57.0 (40.3)	57.2 (40.3)	53.2 (39.5)	<0.0001
CaCO_3_ concentration, mean (SD)	145 (109)	145 (110)	134 (108)	<0.0001
Mg concentration, mean (SD)	5.04 (3.85)	5.04 (3.84)	5.01 (4.02)	0.0047
Greenspace percentage in 300 m, mean (SD)	36.2 (23.7)	36.2 (23.8)	35.1 (22.2)	0.0347
Greenspace percentage in 1,000 m, mean (SD)	46.0 (22.0)	46.0 (22.1)	44.9 (21.0)	<0.0001

The overall baseline characteristics of the included participants were as follows: 135,823 (51.2%) were female, with a mean recruitment age of 56.30 years; 95.2% were White, and the mean body mass index (BMI) was 27.30 kg/m^2^ (*SD* = 4.82). For greenspace exposure, the mean percentage was 36.2 (*SD* = 23.7) in the 300 m buffer and 46.0 (*SD* = 22.0) in the 1,000 m buffer, both treated as continuous variables in all primary analyses. Regarding environmental co-exposures, the mean 24-h sound level, PM_2.5 − 10_ μm concentration, and water hardness (USGS classification) were 56.1 (*SD* = 4.28), 6.42 (*SD* = 0.880), and 2.48 (*SD* = 1.30), respectively.

Between-group comparisons revealed significant differences in demographic, socioeconomic, lifestyle, health, and environmental co-exposure profiles between the depression and no depression developed groups. Compared with participants without depression, those who developed depression had a higher proportion of females, younger individuals, lower university education rates, higher unemployment rates, higher proportions of underweight and obese individuals, higher current smoking rates, more individuals with daily sleep duration <7 h, higher prevalences of diabetes, hypertension, and cancer, poorer self-rated health, higher sound levels, lower water hardness and calcium concentrations, and significantly lower greenspace percentages in both buffers. Detailed between-group comparisons are provided in Table 2.

### 3.2 Association between greenspace and incident depression

During a mean follow-up period, a total of 10,766 (4.06%) participants experienced incident all-cause depression among 265,311 included individuals. The incidence rates of depression progressively decreased across increasing levels of greenspace percentage in both the 300 m and 1,000 m buffers. The associations between residential greenspace and incident depression across all six progressively adjusted Cox models are presented in Table 1.

Across the six progressively adjusted Cox proportional hazards models, higher greenspace exposure was generally associated with a lower risk of incident depression for both the 300 m and 1,000 m buffers. For greenspace within 300 m, each unit increase in greenspace percentage was associated with a lower risk of depression in the fully adjusted model (Model 6: *HR* = 0.9988, 95% *CI*: 0.9979–0.9996; *P* = 0.0049). Similar results were observed for greenspace within 1,000 m, with somewhat stronger associations than those for the 300 m buffer (Model 6: *HR* = 0.9983, 95% *CI*: 0.9974–0.9992; *P* = 0.0003). Overall, these findings indicate that greater residential greenspace exposure was associated with a lower risk of incident depression.

When greenspace was modeled as a continuous variable, restricted cubic spline analyses showed evidence of non-linearity for both buffer sizes (Figure 2). For greenspace within 300 m, the association with incident depression was non-linear (*P*-non-linearity <0.0001), with the exposure–response curve suggesting a slight increase in risk at lower levels of greenspace followed by a progressive decline at higher levels of exposure. For greenspace within 1,000 m, there was also evidence of non-linearity (*P*-non-linearity = 0.0143). The corresponding curve showed modest fluctuation at lower to mid-range exposure levels, followed by a decline in depression risk at higher levels of greenspace. Taken together, the spline analyses suggest that higher greenspace exposure was associated with lower depression risk, although the association was non-linear rather than strictly linear.

**Figure 2:**
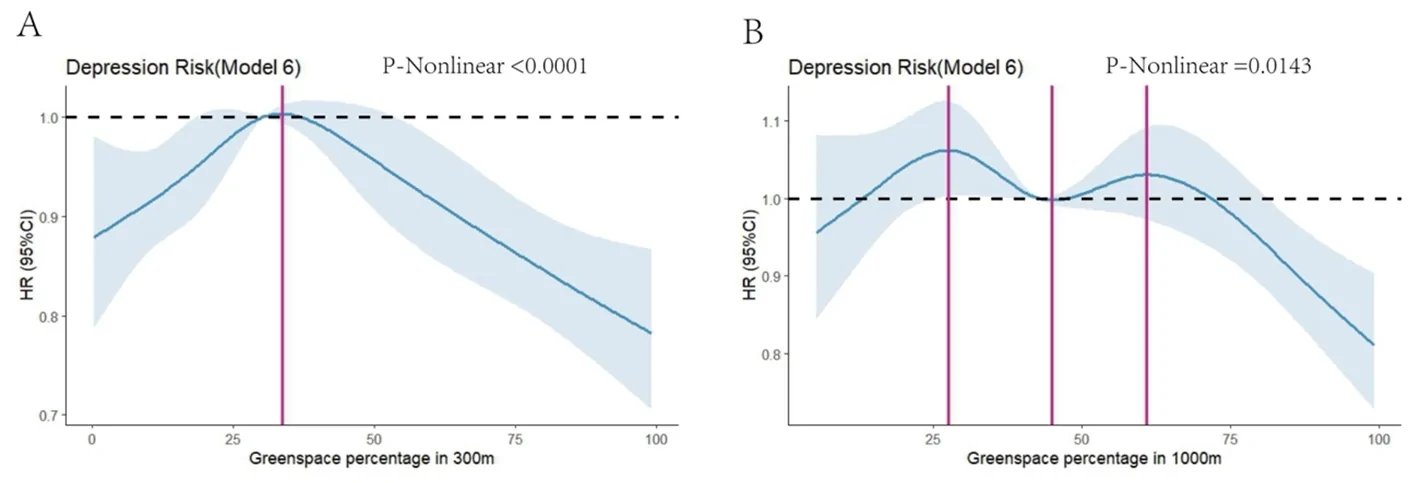
Restricted cubic spline for testing the hypothesis of non-linear correlation between **(A)** greenspace percentage in 300 m, **(B)** greenspace percentage in 1,000 m and depression incidence.

Subgroup analyses are shown in Figure 3. The inverse associations between greenspace and incident depression were generally observed across most subgroups for both the 300 m and 1,000 m buffers. Overall, the subgroup results were broadly consistent with the main analyses, supporting the robustness of the observed association between higher residential greenspace exposure and lower depression risk.

**Figure 3:**
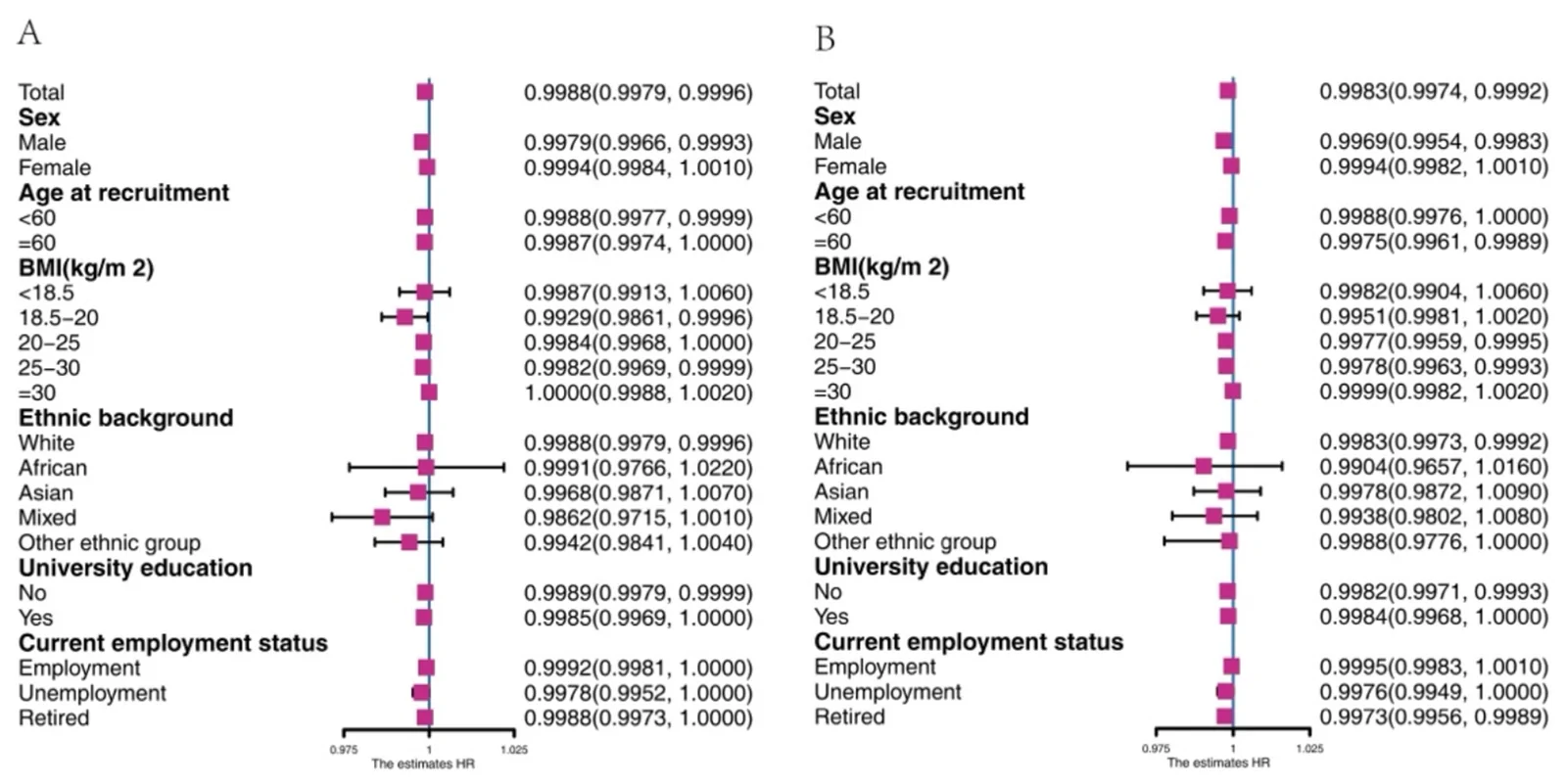
Subgroup analysis between **(A)** greenspace percentage in 300 m, **(B)** greenspace percentage in 1,000 m and depression incidence.

### 3.3 Mediation analysis

Mediation analysis was performed to explore the potential mediating roles of sleep duration and nighttime sound level in the inverse association between residential greenspace exposure and incident depression, with separate evaluations conducted for greenspace percentage within 300 m and 1,000 m residential buffers (Figures 4A–D). For the 300 m buffer, sleep duration exerted a significant partial mediating effect, with an indirect effect (IE) of 2.73 × 10^−6^ (*P* < 0.0001) accounting for 5.28% of the total association (*P* < 0.0001) alongside a significant direct effect (DE) of 4.90 × 10^−5^ (*P* < 0.0001), while nighttime sound level also showed a significant partial mediating effect, with an IE of 8.20 × 10^−7^ (*P* = 0.04) contributing 1.71% of the total association (*P* = 0.04) and a significant DE of 4.89 × 10^−5^ (*P* < 0.0001). For the 1,000 m buffer, sleep duration remained a consistent significant partial mediator, with an IE of 3.63 × 10^−6^ (*P* < 0.0001) accounting for 4.86% of the total association (*P* < 0.0001) and a significant DE of 7.11 × 10^−5^ (*P* < 0.0001), whereas nighttime sound level did not demonstrate a statistically significant mediating effect, with an IE of 1.27 × 10^−7^ (*P* = 0.16), a mediated proportion of 0.18% (*P* < 0.0001), and a non-significant DE of 7.08 × 10^−5^ (*P* = 0.16). Collectively, these findings confirmed that sleep duration partially and significantly mediated the protective effect of residential greenspace against incident depression across both immediate (300 m) and broader (1,000 m) neighborhood buffers, while nighttime sound level only exerted a significant mediating role in the 300 m immediate residential environment, and the relatively small overall mediated proportions indicated that additional unmeasured biological, psychosocial or environmental pathways may also contribute to the observed inverse association between greenspace exposure and depression risk.

**Figure 4:**
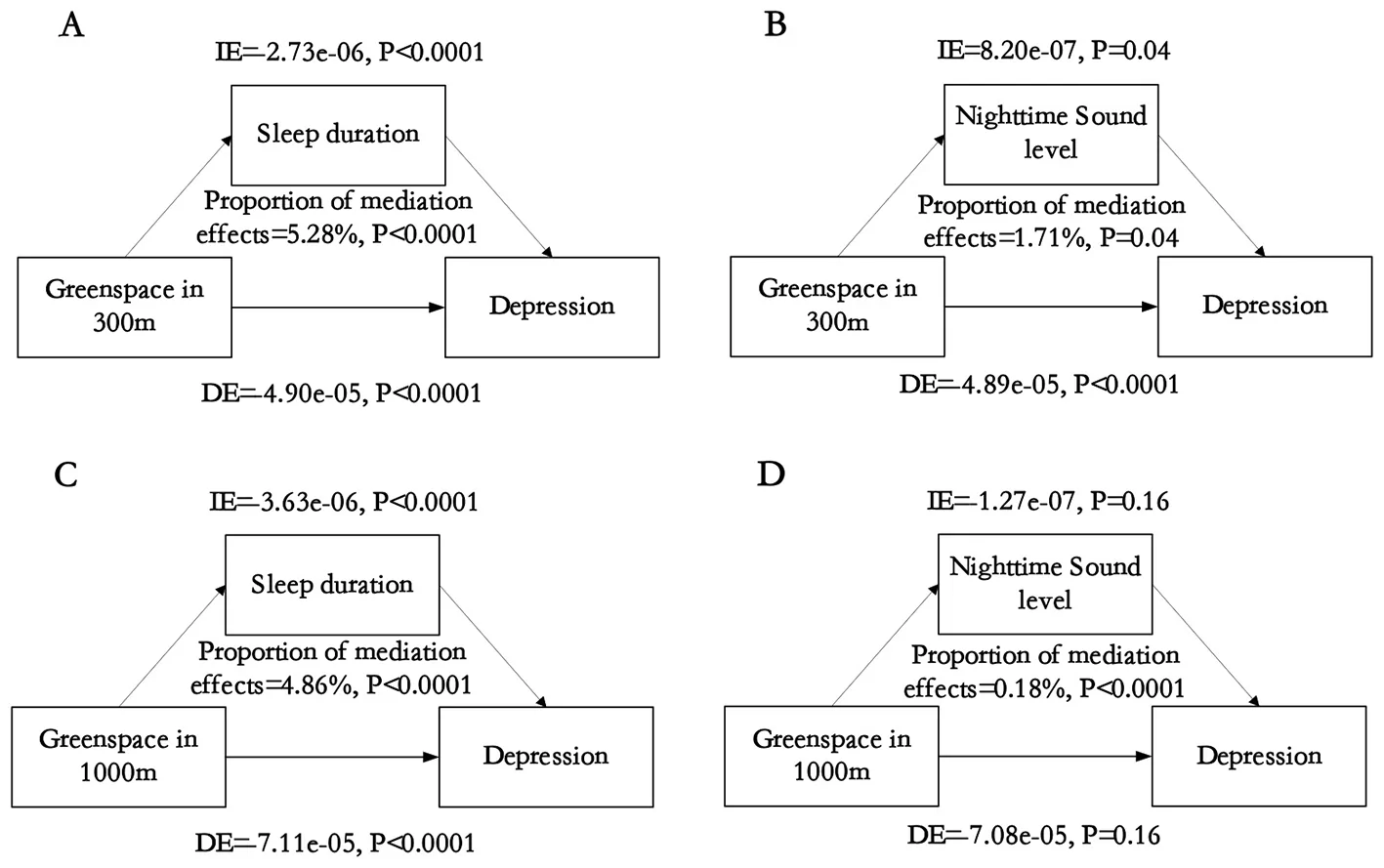
Mediation analysis between **(A)** greenspace percentage in 300 m and sleep duration, **(B)** greenspace percentage in 300 m and nighttime sound level, **(C)** greenspace percentage in 1,000 m and sleep duration, **(D)** greenspace percentage in 1,000 m and nighttime sound level.

## 4 Discussion

Depression remains a leading global cause of disability and a major public health priority, with high prevalence, persistent functional impairment, and substantial socioeconomic and clinical burdens (1, 3, 4). As individual-level preventive interventions often lack scalability, modifiable environmental factors have become critical targets for population-level mental health promotion. Residential greenspace is increasingly recognized as a key protective environmental factor for mental health; however, existing longitudinal evidence is limited by single-scale exposure assessment, insufficient adjustment for environmental co-exposures, simplified dose–response modeling, and incomplete investigation of mediating pathways (6, 12, 22, 23). The present large-scale prospective cohort study addressed these gaps by examining the independent association between residential greenspace (300 m and 1,000 m buffers) and incident all-cause depression, with comprehensive adjustment for sociodemographic, lifestyle, clinical, and environmental confounders, as well as formal mediation analyses of sleep duration and nighttime sound level.

Our primary finding—that higher greenspace percentage within both buffers was associated with a significantly reduced risk of incident depression—is highly consistent with core conclusions from existing population-based longitudinal studies linking increased greenness exposure to improved mental health and lower risk of affective disorders (12, 24). Expressing the associations per 10-percentage-point increase in residential greenspace provides a more interpretable estimate of their potential public health relevance. Although the observed effect size was modest, this may reflect the complex nature of depression, which is influenced by multiple genetic, socioeconomic, behavioral, and psychosocial factors. Therefore, the independent contribution of any single environmental exposure is likely to be relatively small. Nevertheless, even modest reductions in individual risk may have important population-level implications given the widespread exposure to residential greenspace. The large UK Biobank cohort also enabled more precise estimation of these modest but consistent associations. These findings suggest that greenspace should not be considered a standalone intervention for depression prevention, but rather as one potentially modifiable environmental component that may contribute to broader public health and urban planning strategies.

Compared with previous studies, the present study strengthens the existing evidence through several methodological advantages. First, residential greenspace was objectively assessed using two commonly adopted spatial buffers (300 m and 1,000 m), enabling evaluation of associations across different residential scales and facilitating comparison with previous studies that adopted similar buffer sizes (13, 14). Second, unlike studies adjusting only for sociodemographic and lifestyle confounders, we comprehensively controlled for multiple environmental co-exposures including sound levels, coarse particulate matter, and water quality indicators, minimizing residual confounding and strengthening causal interpretability (20). Third, the fully adjusted inverse association remained robust across all sequential models, providing more reliable evidence for the independent protective effect of greenspace than studies with limited covariate adjustment (6).

Restricted cubic spline analyses revealed a significant non-linear dose–response relationship between continuous greenspace exposure and depression risk, which refines and extends findings from prior studies that often treated greenspace as categorical or assumed linearity (6, 8, 12). Our data showed that depression risk remained relatively stable at low-to-moderate greenspace levels but declined progressively at higher exposure levels, indicating a potential threshold effect. This represents an important methodological advance by characterizing the continuous exposure–response pattern, which provides quantitative insights for evidence-based urban planning that cannot be derived from categorical or linear analyses alone (8, 9). This innovation enhances the translational value of our findings for public health and urban design practices.

A further novel contribution is the simultaneous investigation of sleep duration and nighttime sound level as dual mediators, addressing fragmented mediation evidence in previous literature. Consistent with existing mechanistic frameworks linking green environments to sleep quality and noise reduction (17, 23), our results confirmed that sleep duration exerted a significant partial mediating effect at both spatial scales, while nighttime sound level mediated the association only in the 300 m immediate residential buffer. Compared with prior studies examining only one mediator or a single spatial scale, this dual-mediator, dual-scale design clarifies the spatial specificity of mediating pathways: proximal greenspace may directly reduce nighttime noise disturbance, whereas greenspace at broader scales may primarily benefit mental health through improved sleep. The small total mediated proportions also align with existing theoretical models suggesting multiple unmeasured pathways (e.g., stress reduction, physical activity, social cohesion, air pollution mitigation) (5, 10), highlighting the need for integrated multi-mediator research.

From a methodological perspective, this study possesses three key strengths that elevate its evidence level relative to most existing research in environmental psychiatry. First, the prospective cohort design establishes clear temporal ordering of greenspace exposure preceding depression onset, minimizing reverse causation bias that limits cross-sectional studies (3, 5). Second, the long median follow-up period (>10 years) enables capture of the cumulative effects of chronic environmental exposure, which is critical for studying slowly progressive mental disorders like depression (3, 17). Third, the large sample size (>265,000 participants) ensures high statistical power for subgroup and mediation analyses, while the six-stage sequential adjustment framework reduces confounding from a comprehensive set of factors (4, 17). Collectively, these advantages make our findings more generalizable and causally informative than those from small-scale, short-term, or cross-sectional investigations.

Several limitations should be noted, many of which are shared with existing environmental epidemiological studies (6, 9, 10, 19). First, greenspace, sleep duration, and nighttime sound level were assessed only at baseline; therefore, changes in residential location, lifestyle, or environmental conditions during the approximately 10-year follow-up period could not be fully captured, which may have resulted in exposure misclassification. Second, the UK Biobank greenspace metrics reflect overall greenspace availability within residential buffers and cannot differentiate specific vegetation types, such as tree canopy and grass cover. Previous studies have suggested that vegetation types and spatial configurations may have heterogeneous associations with mental health outcomes (13). Therefore, our findings should be interpreted as associations with overall residential greenspace rather than specific vegetation characteristics. In addition, validated measures of urbanicity and broad climatic region were not available in the UK Biobank environmental variables used in this study. Although the UK has a relatively homogeneous temperate maritime climate, residual confounding related to urbanicity, geographic location, or local environmental conditions cannot be completely excluded. Future studies incorporating more detailed spatial environmental data are needed to further clarify the roles of greenspace characteristics and broader environmental contexts in depression risk. Third, we did not explore subgroup-specific differences in mediation effects (e.g., by age, sex, socioeconomic status, or chronic disease), which could identify vulnerable populations that benefit most from green interventions. Fourth, the predominantly White study sample limits generalizability to other ethnic groups. Additionally, UK Biobank participants are generally healthier and more socioeconomically advantaged than the general population, suggesting potential healthy volunteer bias. This limitation should be considered when interpreting the population-level implications of our findings. Future research should adopt time-varying exposure assessment, higher-resolution vegetation classification, network-based exposure metrics, multi-ethnic and multi-center designs, and advanced multi-mediation models to further disentangle the biological and psychosocial pathways linking residential environments to depression risk (2, 8, 19).

## 5 Conclusion

In conclusion, this large-scale prospective cohort analysis confirms that the combined interventions of increased residential greenspace accessibility, prolonged sleep duration, and reduced nighttime sound level are associated with a significantly lower risk of incident depression, and this association is independent of potential confounders such as demographic characteristics and lifestyle factors. The conclusions of this study are mutually corroborated with the research outcomes of other scholars, and also provide evidence-based support for targeted public health interventions: a more mental health-friendly living environment can be constructed by optimizing neighborhood greening environments, guiding individuals to prolong sleep duration, and controlling nighttime environmental sound levels. Future research should further conduct multicenter, cross-ethnic environmental mental health studies, and combine methods such as time-varying analysis and multi-mediation models to deeply explore the molecular mechanisms underlying the effects of environmental factors on mental health. These efforts will provide scientific support for the development of more precise preventive strategies for depression and the improvement of population mental health levels.

## Data Availability

The original contributions presented in the study are included in the article/Supplementary material, further inquiries can be directed to the corresponding author/s.
