# Supplementary material for: Association of residential greenspace with incident depression: a prospective cohort study
Source: Front Public Health. 2026 Sep 30;14:1897674. doi: 10.3389/fpubh.2026.1897674 (PMC13661952; doi:10.3389/fpubh.2026.1897674)
Supplement: Supplementary file 1 [file Data_Sheet_1.pdf]

Supplementary Material

1 Supplementary Tables

Supplementary Table S1. Participants in the UK Biobank were recruited at 40–69 years of age at baseline. Given the restricted age range of the cohort, age was included as a categorical covariate in the fully adjusted Cox proportional hazards models in the primary analyses. To evaluate whether the categorization of age influenced the estimated associations between residential greenspace exposure and incident depression, we conducted a sensitivity analysis by modeling age as a continuous variable. The associations between greenspace exposure within 300 m and 1000 m residential buffers and incident depression remained largely consistent with the primary analyses, with comparable effect estimates and similar directions of associations, supporting the robustness of the findings.

Supplementary Table S1. Sensitivity analysis of the association between greenspace exposure and incident depression using age as a continuous variable.

	Greenspace percentage in 300m, HR (95%CI)	$P_{Cox\text{-}regression}$	Greenspace percentage in 1000m, HR (95%CI)	$P_{Cox\text{-}regression}$
Model 1	0.9789 (0.9713, 0.9880)	<0.0001	0.9752 (0.9667, 0.9841)	<0.0001
Model 2	0.9772 (0.9687, 0.9853)	<0.0001	0.9696 (0.9612, 0.9780)	<0.0001
Model 3	0.9851 (0.9764, 0.9930)	0.0003	0.9811 (0.9721, 0.9910)	<0.0001
Model 4	0.9930 (0.9851, 1.0020)	0.1080	0.9910 (0.9821, 0.9990)	0.0418
Model 5	0.9930 (0.9851, 1.0010)	0.1046	0.9920 (0.9821, 0.9990)	0.0449
Model 6	0.9891 (0.9812, 0.9980)	0.0133	0.9851 (0.9755, 0.9940)	0.0011

Data are presented as Hazard Ratios (HR) and 95% Confidence Intervals (CI) per 10% increment in greenspace percentage.

Model 1: Sex, Age at recruitment, Ethnic background and BMI.

Model 2: Variables in Model 1 plus Employment status and University education.

Model 3: Variables in Model 2 plus Alcohol drinker status, Smoking status and Sleep duration.

Model 4: Variables in Model 3 plus Diabetes, Hypertension, Cancer and Overall health rating.

Model 5: Variables in Model 4 plus Coffee intake, Tea intake, IPAQ activity group, 24 hour-Sound level, Daytime sound level and Nighttime sound level.

Model 6: Variables in Model 5 plus PM2.5-10um, water hardness (USGS classification), Ca concentration, $CaCO_3$ concentration and Mg concentration.

Abbreviations: IPAQ: International Physical Activity Questionnaire; USGS: United States Geological Survey (Water hardness classification criteria); PM: Particulate Matter.

Supplementary Table S2. The table summarizes the definition, data sources, and derivation procedures of residential greenspace exposure variables used in this study.

Supplementary Table S2. Sources and definitions of greenspace and environmental exposure variables derived from the UK Biobank.

FieldID	Field	Notes	Link
24500	Greenspace percentage, buffer 1000m	The percentage of the home location buffer classed as 'Greenspace', as a proportion of all land-use types, and with home location data buffered at 1000m. Land use data were obtained from the Generalized land use database for England (GLUD) 2005 data at the 2001 Census Output Area level. Each home location polygon was then allocated an area weighted mean of the land use percentage coverage for the 2001 Census Output Areas intersecting the home location buffer. Data are only available for participants whose home location was in England. Further details of the methods used to derive these data can be found in Resource 15374, where this field is derived from variables referred to as gs1000_0 and gs1000_1.	http://biobank.ndph.ox.ac.uk/ukb/field.cgi?id=24500
24503	Greenspace percentage, buffer 300m	The percentage of the home location buffer classed as 'Greenspace', as a proportion of all land-use types, and with home location data buffered at 300m. Land use data were obtained from the Generalized land use database for England (GLUD) 2005 data at the 2001 Census Output Area level. Each home location polygon was then allocated an area weighted mean of the land use percentage coverage for the 2001 Census Output Areas intersecting the home location buffer. Data are only available for participants whose home location was in England. Further details of the methods used to derive these data can be found in Resource 15374, where this field is derived from variables referred to as gs300_0 and gs300_1.	http://biobank.ndph.ox.ac.uk/ukb/field.cgi?id=24503
24020	Average daytime sound level of noise pollution	LDay (day equivalent level): Average sound level pressure LAeq over the 12-hour period 07:00 to 19:00.	http://biobank.ndph.ox.ac.uk/ukb/field.cgi?id=24020

24022	Average night-time sound level of noise pollution	LNight (night equivalent level): Average sound level pressure LAeq overnight 23:00 to 07:00.	http://biobank.ndph.ox.ac.uk/ukb/field.cgi?id=24022
24024	Average 24-hour sound level of noise pollution	LDen: (day-evening-night equivalent level): A-weighted Leq noise level measured over the 24 hour period with a 10 decibel penality added to the levels between 23:00 and 07:00.	http://biobank.ndph.ox.ac.uk/ukb/field.cgi?id=24024
24008	Particulate matter air pollution 2.5-10um; 2010	Particulate matter air pollution 2.5-10um; 2010	http://biobank.ndph.ox.ac.uk/ukb/field.cgi?id=24008
21104	Ca concentration	Domestic water calcium concentration.	https://bb30.ndph.ox.ac.uk/ukb/field.cgi?id=21104
21103	CaCO3 concentration	Domestic water calcium carbonate concentration.	https://bb30.ndph.ox.ac.uk/ukb/field.cgi?id=21103
21105	Mg concentration	Domestic water magnesium concentration.	https://bb30.ndph.ox.ac.uk/ukb/field.cgi?id=21105
21100	Water hardness (USGS classification)	United States Geological Survey classification of CaCO3 concentration into four groups: soft water (0-60 mg/L), moderately hard water (60-120 mg/L), hard water (120-180 mg/L) and very hard water (> 180 mg/L).	https://bb30.ndph.ox.ac.uk/ukb/field.cgi?id=21100
